# Impaired mitochondrial medium-chain fatty acid oxidation drives periportal macrovesicular steatosis in sirtuin-5 knockout mice

**DOI:** 10.1038/s41598-020-75615-3

**Published:** 2020-10-27

**Authors:** Eric S. Goetzman, Sivakama S. Bharathi, Yuxun Zhang, Xue-Jun Zhao, Steven F. Dobrowolski, Kevin Peasley, Sunder Sims-Lucas, Satdarshan P. Monga

**Affiliations:** 1 Department of Pediatrics, Children’s Hospital of Pittsburgh of UPMC, University of Pittsburgh School of Medicine, Pittsburgh, PA USA; 2 Department of Pathology, University of Pittsburgh School of Medicine, Pittsburgh, PA USA; 3 Division of Experimental Pathology, Department of Pathology, University of Pittsburgh School of Medicine, Pittsburgh, PA USA; 4 Division of Gastroenterology, Hepatology and Nutrition, Department of Medicine, University of Pittsburgh School of Medicine, Pittsburgh, PA USA; 5 Pittsburgh Liver Research Center, University of Pittsburgh Medical Center, University of Pittsburgh School of Medicine, Pittsburgh, PA USA

**Keywords:** Biochemistry, Physiology, Molecular medicine

## Abstract

Medium-chain triglycerides (MCT), containing C_8_–C_12_ fatty acids, are used to treat several pediatric disorders and are widely consumed as a nutritional supplement. Here, we investigated the role of the sirtuin deacylase Sirt5 in MCT metabolism by feeding Sirt5 knockout mice (Sirt5KO) high-fat diets containing either C_8_/C_10_ fatty acids or coconut oil, which is rich in C_12_, for five weeks. Coconut oil, but not C_8_/C_10_ feeding, induced periportal macrovesicular steatosis in Sirt5KO mice. ^14^C–C_12_ degradation was significantly reduced in Sirt5KO liver. This decrease was localized to the mitochondrial β-oxidation pathway, as Sirt5KO mice exhibited no change in peroxisomal C_12_ β-oxidation. Endoplasmic reticulum ω-oxidation, a minor fatty acid degradation pathway known to be stimulated by C_12_ accumulation, was increased in Sirt5KO liver. Mice lacking another mitochondrial C_12_ oxidation enzyme, long-chain acyl-CoA dehydrogenase (LCAD), also developed periportal macrovesicular steatosis when fed coconut oil, confirming that defective mitochondrial C_12_ oxidation is sufficient to induce the steatosis phenotype. Sirt5KO liver exhibited normal LCAD activity but reduced mitochondrial acyl-CoA synthetase activity with C_12_. These studies reveal a role for Sirt5 in regulating the hepatic response to MCT and may shed light into the pathogenesis of periportal steatosis, a hallmark of human pediatric non-alcoholic fatty liver disease.

## Introduction

The sirtuin deacylase Sirt5 is one of seven mammalian sirtuins that remove lysine acylation post-translational modifications (PTMs) from cellular proteins. Sirt5 is unique in the sirtuin family for its specificity in removing succinyl, glutaryl, and malonyl groups from lysine residues^1–3^. While its main site of action is the mitochondria, Sirt5 is also the only sirtuin shown to localize to peroxisomes^4^. In liver mitochondria, Sirt5 desuccinylation positively regulates fatty acid oxidation (FAO) and ketogenesis, the process by which FAO-derived acetyl-CoA is converted to ketone bodies for secretion to the periphery^3,5^. Interestingly, in the peroxisome, which possesses a parallel FAO pathway to that found in mitochondria, Sirt5 negatively regulates the key FAO protein acyl-CoA oxidase-1 (ACOX1)^4^. Based on these studies, it would be predicted that deleting Sirt5 would suppress mitochondrial FAO while promoting peroxisomal FAO. However, this has not been directly tested. Here, we investigated the effect of Sirt5 deletion on FAO in mice maintained on a high-fat coconut oil diet. Unlike the lard-based high-fat diets typically used in rodent studies, coconut oil has very little long-chain fatty acids. Rather, coconut oil is rich in medium-chain triglyercides (MCT).

MCT, defined as triglycerides containing fatty acids C_8_ to C_12_ in length, have a broad significance to human health. MCT are a key component of nutritional formulas given to premature infants^6^. MCT are also used to treat long-chain fatty acid oxidation disorders, gastrointestinal disorders, and epilepsy, among others^7–9^. Recently, consumption of MCT as a nutritional supplement has increased tremendously due to the popularity of ketogenic diets for weight loss^10^. Medium-chain fatty acids released from MCT in the gut are poor substrates for re-synthesis of triglycerides within enterocytes, and as a result they are absorbed into the portal vein as free acids and send to the liver for disposal. Less than 1% of the ingested dose of MCT will reach peripheral circulation^11^. Consumption of large doses of MCT exposes hepatocytes to high concentrations of free medium-chain fatty acids, which must be either immediately catabolized or else elongated to a chain length that can be stored as triglyceride. This contrasts with long-chain fatty acids released from long-chain triglycerides (LCT), which are re-esterified into triglycerides, packed into chylomicrons, and trafficked from the gut through the lymphatic system and into peripheral circulation.

Not all MCT-based nutritional products contain the same composition of fatty acids. Coconut oil is primarily C_12_ (50%), but also contains minor amounts of C_14_, C_10_, and C_8_^12^. Other MCT products contain either pure C_8_ or a mixture of C_8_ and C_10_ obtained from fractionating coconut oil. C_8_/C_10_ may undergo a different route of metabolism than C_12_ due to the substrate specificity and intracellular localization of the acyl-CoA synthetase enzymes responsible for conjugating free fatty acids to acyl-CoAs, which are the biologically active form of fatty acids in the cell. C_8_, and to lesser extent C_10_, can diffuse into the mitochondrial matrix where they are activated into acyl-CoAs by the medium-chain acyl-CoA synthetases (ACSMs)^13^. C_12_ cannot cross the mitochondrial membrane and is a poor substrate for ACSMs^13^. C_12_ is activated to C_12_-CoA by the long-chain acyl-CoA synthetases (ACSLs), which reside on the outer mitochondrial membrane, plasma membrane, ER, and the peroxisomal membrane^14^. In keeping with this, C_12_ can be catabolized by both mitochondrial and peroxisomal β-oxidation pathways^15^. C_12_ is also metabolized by a minor pathway known as ω-oxidation in which C_12_ is hydroxylated by cytochrome P450 enzymes in the endoplasmic reticulum, converted to a dicarboxylic acid in the cytosol, and finally chain-shortened in the peroxisome^16^. While peroxisomes can chain-shorten long-chain fatty acids down to C_6_^17^ and DCAs as far as C_4_^18^, the capacity of the peroxisome to catabolize exogenous C_8_/C_10_ is limited by the fact that all known ACSMs reside in the mitochondrial matrix^19^.

In rodent models, both C_8_ and C_12_ feeding have been linked to hepatic steatosis^15,20^. However, other studies have indicated either no fat accumulation or even a reversal of pre-existing fatty liver, leading to the suggestion that MCT may have utility for treating the metabolic syndrome^21,22^. Understanding the molecular mechanisms involved in hepatic adaptation to MCT is critical for determining the impact of this supplement on human health. Because of Sirt5’s dual localization to mitochondria and peroxisomes and its known opposite regulation of FAO enzymes in these compartments, we used Sirt5 knockout mice to interrogate the role of this lysine deacylase on the hepatic response to dietary C_8_/C_10_ versus coconut oil. Our results implicate Sirt5 as an important regulator of C_12_ disposal in the liver.

## Results

### Sirt5KO mice develop hepatic macrovesicular steatosis on coconut oil diet

**Figure 1:**
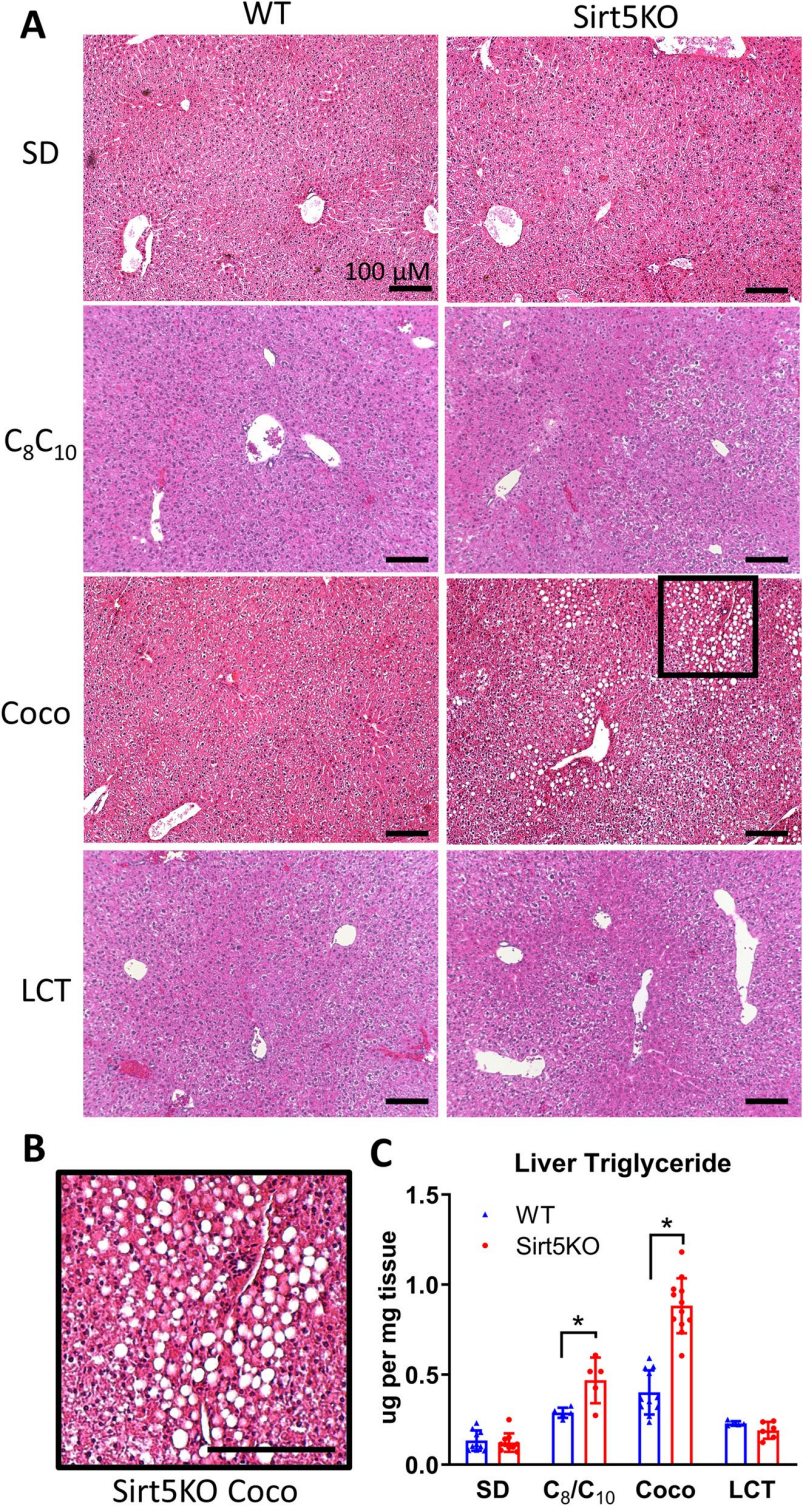
Sirt5KO mice develop hepatic macrovesicular steatosis on coconut oil diet. **(A)** Representative hematoxylin and eosin liver tissue staining from wild-type and Sirt5KO mice maintained on four different diets for five weeks: standard diet (SD), high-fat C_8_/C_10_ diet, high-fat coconut oil diet (Coco), or high-fat long-chain triglyceride (LCT) diet. The relative composition of these diets is illustrated in Supplemental Fig. 1. Fat droplets were visible in only coconut-fed Sirt5KO mice. Scale bar, 100 µM. **(B)** Inset from coconut-fed Sirt5KO liver in panel **(A)**, showing lipid droplets at a higher resolution. **(C)** Liver triglyceride content of mice maintained on the four diets for five weeks. N = 6 for C_8_/C_10_ and LCT diets, and N = 10 for coconut and SD diets. There was no gender difference in triglyceride accumulation; all groups contained half males and half females, age 6–8 weeks at time of diet onset. *P < 0.01 Sirt5KO versus wild-type controls.

**Figure 2:**
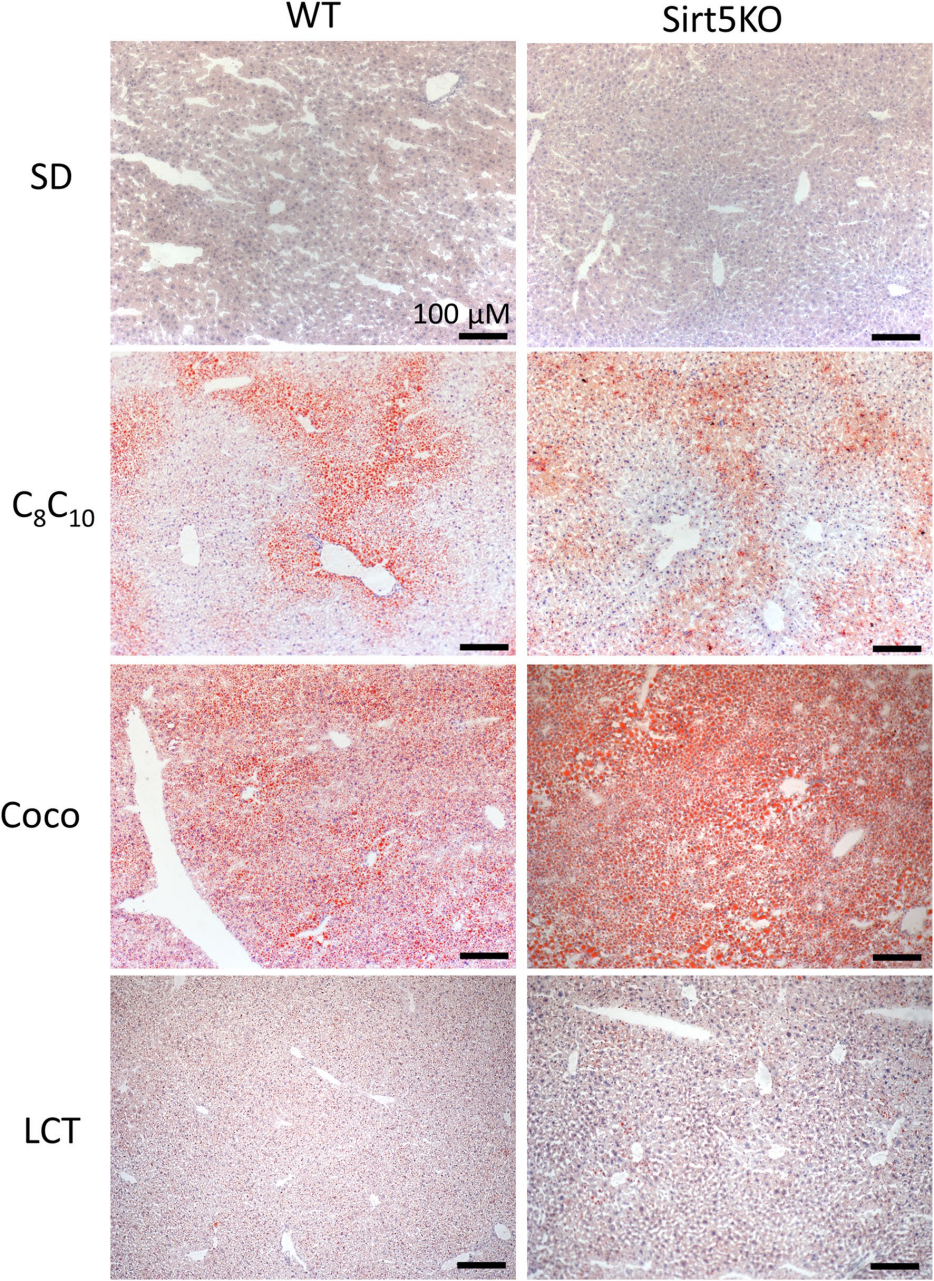
Oil-Red-O staining reveals steatosis in both C_8_/C_10_ and coconut-fed mice. Representative Oil-Red-O stained liver tissue from wild-type (WT) and Sirt5KO mice on four different diets: standard diet (SD), C_8_/C_10_ diet, coconut oil diet (Coco), and long-chain triglyceride diet (LCT). Scale bar, 100 µM.

To study the role of Sirt5 in regulating the hepatic response to acute high-fat feeding, we placed Sirt5KO and wild-type control mice on three high-fat diets containing different chain-lengths of fatty acids: (1) a diet containing 60% C_8_ and 40% C_10_; (2) a coconut-oil diet containing primarily C_12_; and (3) a long-chain triglyceride (LCT) diet containing primarily C_16_ and C_18_ fatty acids. The relative composition of these diets appears in Supplemental Fig. 1. A low-fat standard diet (SD) served as control. After five weeks on the high-fat diets, liver tissue was collected and analyzed for signs of steatosis. In H&E-stained liver sections, macrovesicular steatosis was noted only in Sirt5KO livers from mice maintained on the coconut oil diet (Fig. 1a,b). This was observed in both genders of Sirt5KO mice. While the C_8_/C_10_ diet did not induce overt macrovesicular steatosis, increased microvesicular lipid deposition was visible in liver of both genotypes upon Oil-Red-O staining (Fig. 2). The intensity of the Oil-Red-O staining was even greater with coconut oil feeding. Little or no Oil-Red-O staining was visible in livers from mice fed LCT for five weeks. Together, the H&E and Oil-Red-O stains indicated that fat accumulated in the order of coconut >  > C_8_/C_10_ >  > LCT for both genotypes of mice, with the lipid accumulation progressing to the point of macrovesicular steatosis only in Sirt5KO mice on coconut oil diet. This was supported by a biochemical analysis of liver triglyceride content. Both C_8_/C_10_ and coconut oil feeding significantly increased the triglyceride content of liver tissue over that seen in liver from mice maintained on the SD, while LCT had no effect (Fig. 1c). Sirt5 deficiency was associated with significantly increased liver triglycerides for both the C_8_/C_10_ and coconut oil diets (Fig. 1c). Again, both genders were evaluated, and no gender effect was seen for triglyceride accumulation.

### The macrovesicular steatosis in coconut oil-fed Sirt5KO mice is periportal

**Figure 3:**
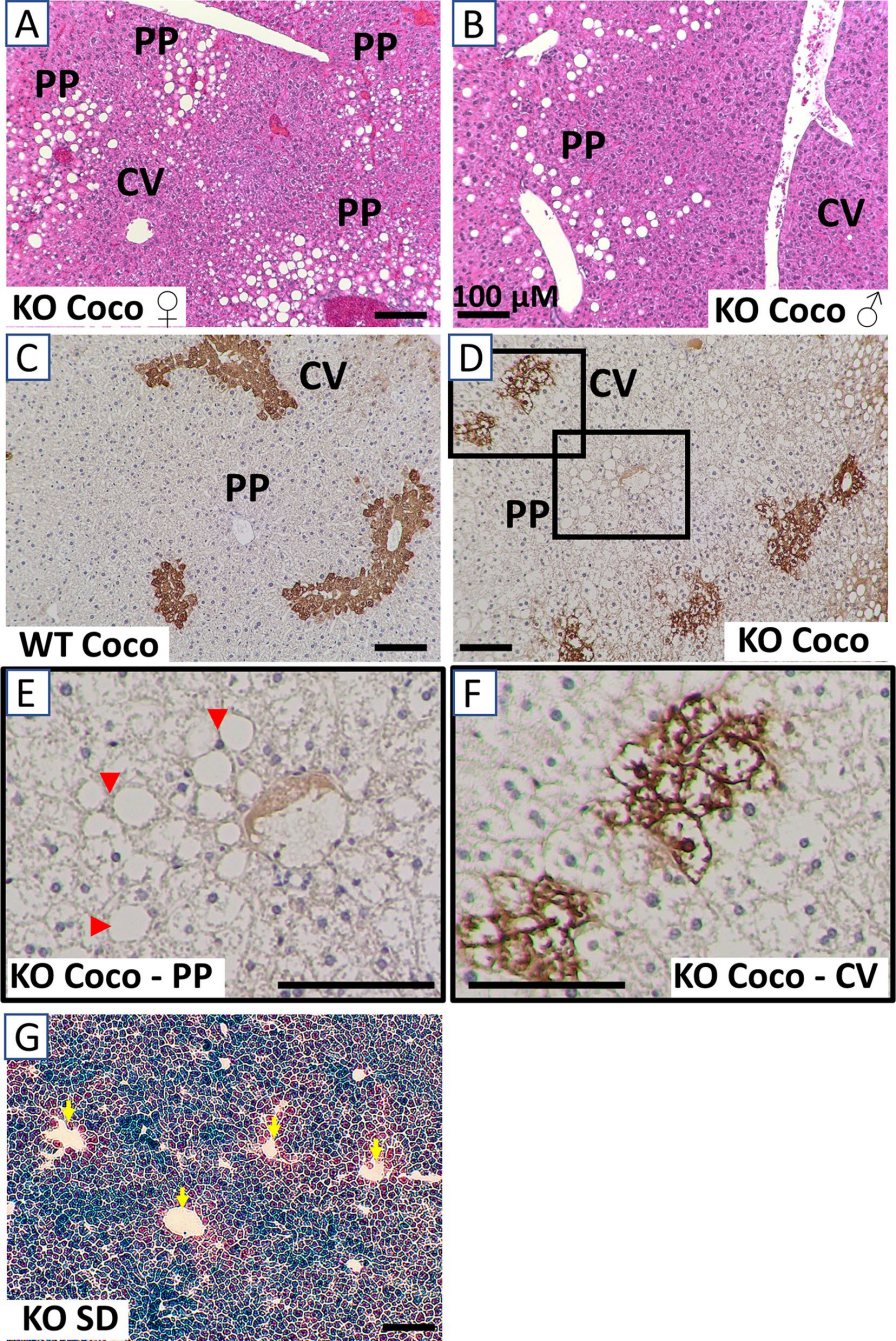
The macrovesicular steatosis in coconut oil-fed Sirt5KO mice is periportal. Both female **(A)** and male **(B)** coconut-fed Sirt5KO mice showed formation of lipid droplets in the periportal (PP) regions but not central vein (CV) regions as gauged by a pathologist’s examination of hematoxylin/eosin-stained liver. **(C)** Liver tissue from coconut-fed wild-type and **(D)** Sirt5KO mice, immunostained for glutamine synthase (GS), a marker of pericentral hepatocytes. **(E,F)** Close-up images of inset areas from **(D)**, showing a periportal region **(E)** and a pericentral region **(F)** from a coconut-fed Sirt5KO liver. Periportal lipid droplets are indicated with arrow heads in **(E)**; no droplets are visible in **(F)**. Note the ballooning degeneration in **(F)**. **(G)** β-galactosidase staining for Sirt5 expression in Sirt5KO liver. The mutant allele in Sirt5KO mice contains a lacZ insert, and β-gal staining can be used as a proxy for Sirt5 expression. Blue staining is less intense around the central veins, indicated with yellow arrowheads. Scale bar, 100 µM.

In adult humans with non-alcoholic fatty liver disease (NAFLD) and NAFLD rodent models induced by chronic LCT feeding, macrovesicular steatosis first appears around the pericentral regions of the liver. In contrast, pediatric NAFLD is characterized by periportal steatosis^23^. Interestingly, while five weeks of coconut oil feeding induced pan-zonal microvesicular steatosis in both wild-type and Sirt5KO livers as visualized by Oil-Red-O staining (Fig. 2), the macrovesicular steatosis in Sirt5KO livers was strictly periportal (Fig. 3a,b). To confirm the periportal localization of the lipid droplets we immunostained the liver tissue for glutamine synthase (GS), a marker of the pericentral zone. No lipid droplets were observed near the GS-positive pericentral zones (Fig. 3c–f). Besides marking the pericentral zones, the GS immunostaining highlighted an altered morphology of the pericentral hepatocytes in Sirt5KO liver that was consistent with ballooning degeneration. Sirt5 has previously been reported to exhibit hepatic zonation, with higher expression in primary mouse hepatocytes isolated from the periportal zone^24^. We confirmed this using beta-galactosidase staining as a proxy for expression of the Sirt5 mutant allele in Sirt5KO mice, which contains a lacZ insert. Pericentral hepatocytes exhibited less staining for the Sirt5 promoter-driven beta-galactosidase enzyme (Fig. 3g).

### Sirt5KO liver has decreased capacity for C_12_ fatty acid β-oxidation (FAO)

**Figure 4:**
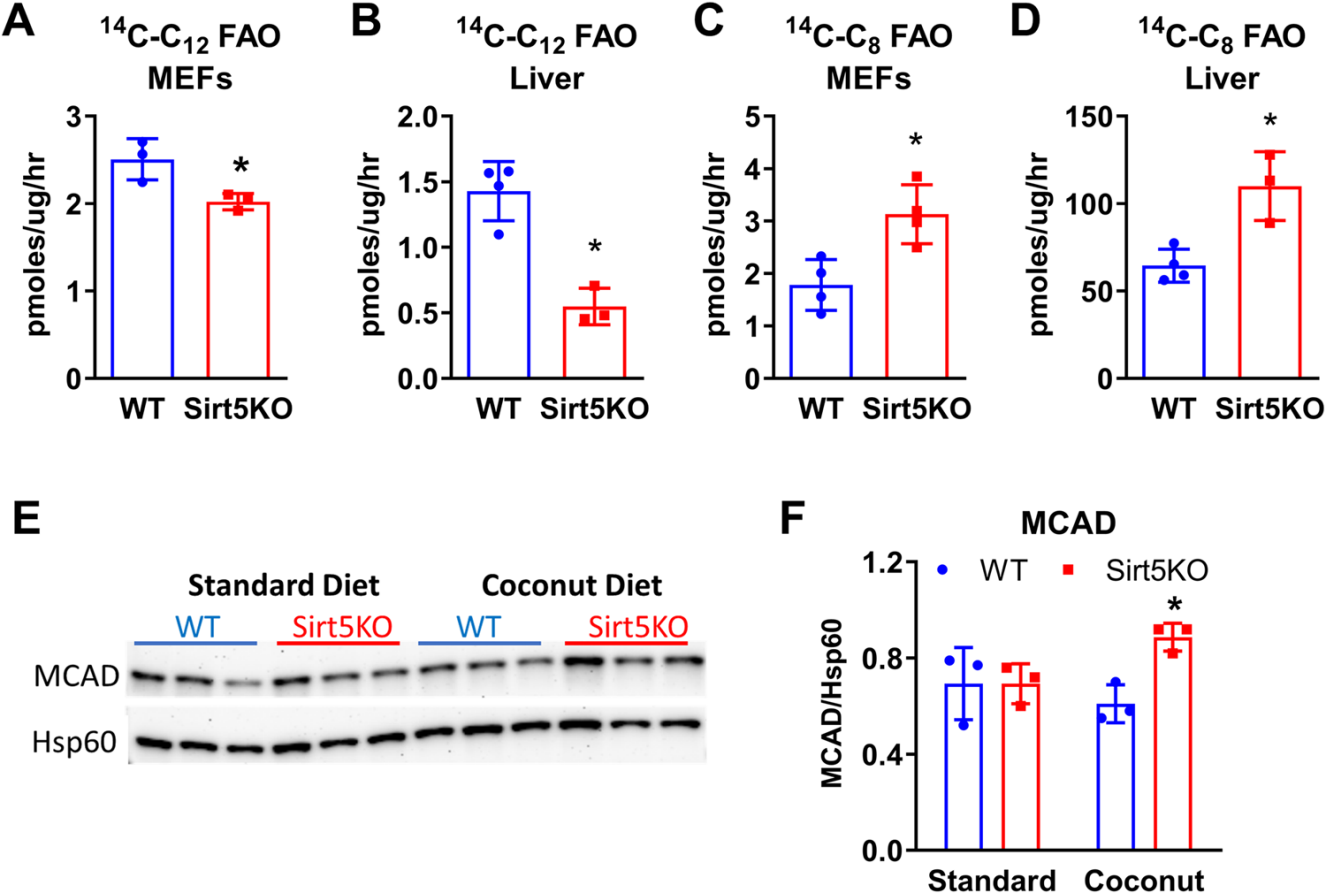
Sirt5KO liver has decreased capacity for C_12_ fatty acid oxidation. **(A)** ^14^C–C_12_ flux to labeled acid-soluble metabolites in wild-type and Sirt5KO mouse embryonic fibroblasts (MEFs); **(B)** ^14^C–C_12_ FAO flux in coconut-fed liver homogenates. **(C)** ^14^C–C_8_ flux to acid-soluble metabolites in MEFs and **(D)** coconut-fed liver homogenates. **(A–D)** are data from N = 3–4 biological replicates. **(E)** Immunoblotting for MCAD in liver from SD and coconut-fed WT and Sirt5KO mice, with heat-shock protein-60 (Hsp60) as a mitochondrial loading control. The uncropped version of these blots appears in Supplemental Fig. 2. **(F)** Densitometric analysis of blot in panel **(E)**, MCAD/Hsp60. *P < 0.05 for Sirt5KO versus wild-type control.

We next sought to investigate the mechanism(s) behind the observed acceleration in lipid storage in Sirt5KO livers during coconut oil feeding. Sirt5KO mice have previously been shown to be deficient in long-chain FAO but have not been evaluated for changes in medium-chain FAO^3^. To interrogate medium-chain FAO we used ^14^C-labeled C_8_ and C_12_ and quantified flux in liver homogenates as well as mouse embryonic fibroblasts (MEFs). Sirt5KO MEFs, cultured in standard medium, showed a significant deficit in ^14^C-C_12_ catabolism (Fig. 4a). In contrast, C_8_ oxidation rates were higher in Sirt5KO MEFs (Fig. 4c). The same pattern was observed in liver homogenates prepared from mice fed coconut oil diet for five weeks (Fig. 4b,d). The higher C_8_ oxidation may be due to higher expression of the C_8_-specific enzyme medium-chain acyl-CoA dehydrogenase (MCAD) in Sirt5KO livers after coconut oil feeding (Fig. 4e,f).

### Peroxisomal FAO is not altered in coconut-fed Sirt5KO mice

**Figure 5:**
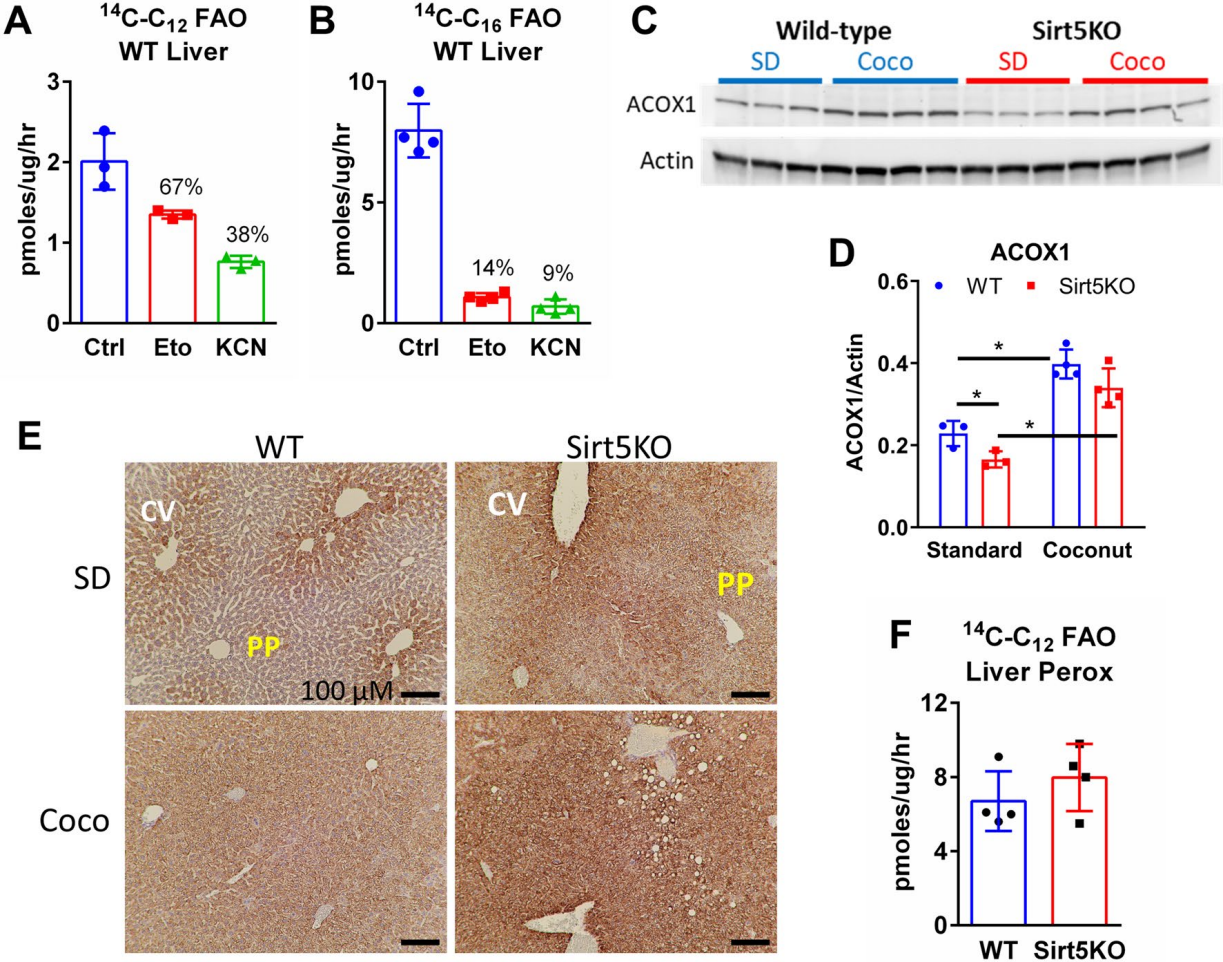
Peroxisomal FAO is not altered in Sirt5KO mice on coconut oil diet. **(A)** Effect of mitochondrial FAO inhibitors on ^14^C–C_12_ and **(B)** ^14^C–C_16_ flux to acid-soluble metabolites. Flux to acid-soluble metabolites was assayed in the presence of either 100 µM of the irreversible Cpt1 inhibitor etomoxir or 2 mM of the respiratory chain inhibitor KCN. **(C)** Immunoblotting for peroxisomal acyl-CoA oxidase-1 (ACOX1) in standard diet (SD, N = 3) and coconut-fed (Coco, N = 4) wild-type and Sirt5KO livers, with β-actin as loading control. The uncropped versions of these blots appear in Supplemental Fig. 3. **(D)** Densitometric analysis of immunoblot in panel C, expressed as ACOX1/β-actin. *P < 0.01 for indicated pairwise comparisons. **(E)** Representative immunostaining of liver tissue from standard diet and coconut-fed with anti-PMP70 antibody, a peroxisomal membrane protein. Note the darker staining around the central vein (CV) compared to periportal (PP) tracts on standard diet. Scale bar, 100 µM. **(F)** ^14^C–C_12_ peroxisomal fatty acid oxidation, measured as KCN-resistant flux to labeled acid-soluble metabolites, N = 4 liver homogenates from coconut-fed mice.

C_12_, the principal fatty acid in coconut oil, is the optimal substrate for the rate-limiting peroxisomal FAO enzyme acyl-CoA oxidase-1 (ACOX1)^25^. In wild-type mouse liver homogenates from animals on standard diet, nearly 40% of the capacity for ^14^C–C_12_ oxidation was resistant to potassium cyanide (KCN) and therefore peroxisomal (Fig. 5a). In contrast, for the long-chain fatty acid (C_16_), peroxisomes contributed only about 10% (Fig. 5b). Because of the strict mitochondrial localization of the acyl-CoA synthetases responsible for activation of C_8_ to C_8_-CoA, C_8_ metabolism is believed to be mitochondria-specific^19^. We therefore reasoned that the low C_12_ oxidation in Sirt5KO liver, but normal/high C_8_ oxidation (Fig. 4), might be due to a specific loss of peroxisomal FAO capacity. Western blotting indicated a significant increase in ACOX1 protein expression in both genotypes of mice when fed coconut oil diet for five weeks (Fig. 5c,d). Immunostaining for the peroxisomal marker protein PMP70 showed that peroxisomes are more enriched in the pericentral zones when mice are maintained on a standard diet but demonstrate a pan-zonal proliferation after five weeks of coconut oil diet (Fig. 5e). This occurred in both wild-type and Sirt5KO mice, but overall, the PMP70 staining was darker in Sirt5KO liver. However, the rate of KCN-resistant (peroxisomal) ^14^C–C_12_ FAO in liver homogenates of coconut oil-fed mice was not significantly different between genotypes (Fig. 5f). This indicates that the reduced capacity for C_12_ oxidation seen in Sirt5KO liver is not attributable to suppressed peroxisomal FAO.

### Fatty acid ω-oxidation is upregulated in Sirt5KO livers

**Figure 6:**
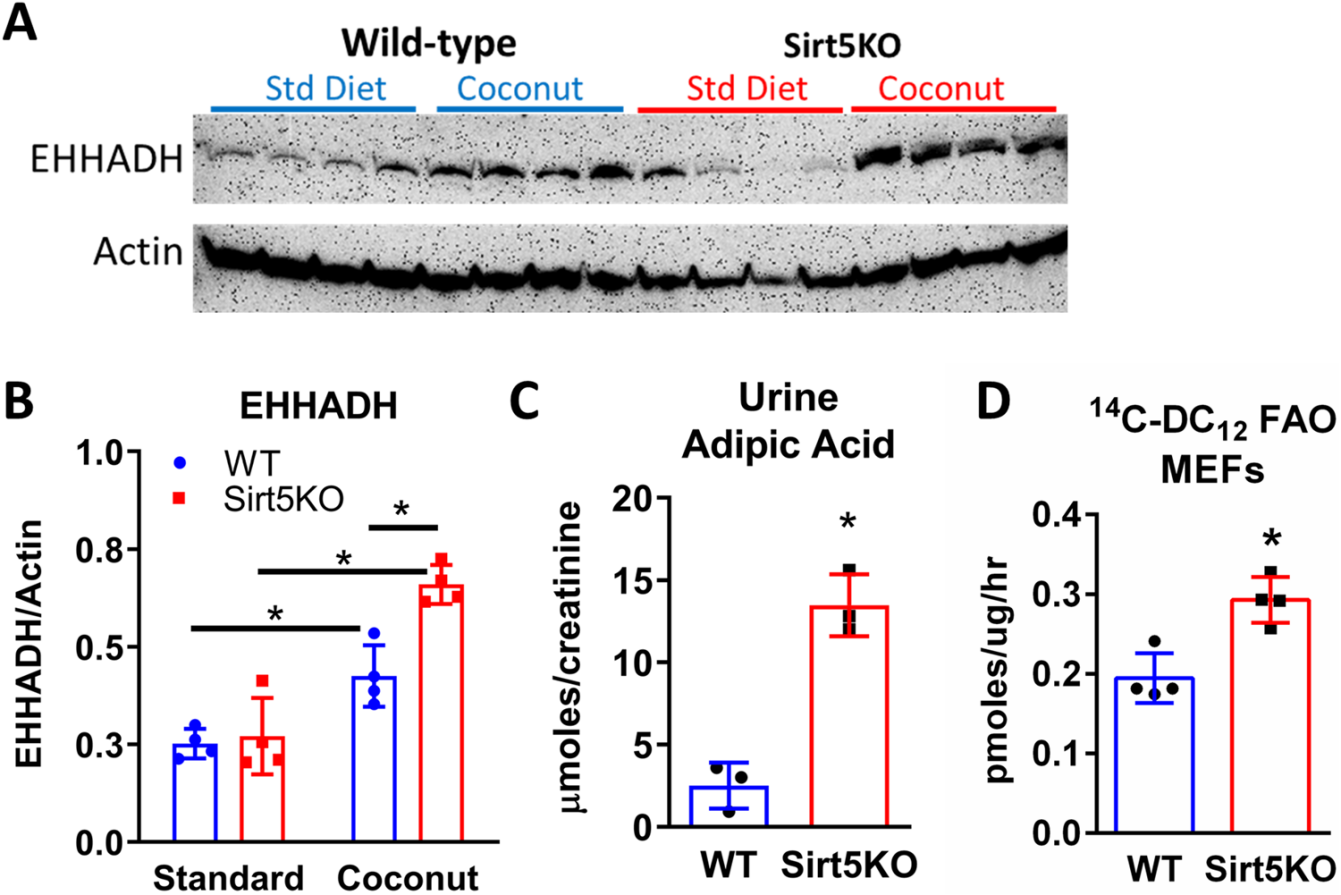
Omega oxidation is upregulated in Sirt5KO livers. **(A)** Immunoblotting for peroxisomal enoyl-CoA hydratase/3-hydroxyacyl-CoA dehydrogenase (EHHADH) in standard diet (SD) and coconut-fed (Coco) wild-type and Sirt5KO livers, N = 4. β-actin served as loading control. The uncropped versions of these blots appear in Supplemental Fig. 4. **(B)** Densitometric analysis of panel **(A)**. *P < 0.01 for indicated pairwise comparisons. **(C)** Urine adipic acid, the peroxisomally-generated product from degradation of the dicarboxylic acids produced by omega oxidation; N = 3 wild-type (WT) and Sirt5KO coconut-fed mice. *P < 0.01. **(D)** Flux of ^14^C-dicarboxylic (DC) C_12_ to labeled acid-soluble metabolites in cultured mouse embryonic fibroblasts (MEFs), N = 4 replicates. *P < 0.05.

In addition to mitochondria and peroxisomes, C_12_ is also the optimal chain length for a minor FAO pathway known as ω-oxidation^16^. ω-oxidation initiates with C_12_ hydroxylation by Cyp4a family members in the ER. Through a poorly characterized pathway, C_12_OH is converted to a dicarboxylic C_12_ fatty acid (DC_12_) and finally catabolized by peroxisomes, with only minimal contribution by mitochondria^26^. Immunoblotting revealed significantly higher protein expression of enoyl-CoA hydratase/3-hydroxy-CoA dehydrogenase (EHHADH), a peroxisomal enzyme known to be required for DC_12_ catabolism^27^, in Sirt5KO liver following five weeks on coconut oil diet (Fig. 6a,b). DC_12_ is chain-shortened to adipic acid which is excreted into the urine. Sirt5KO mice on coconut-oil diet excreted four-fold more adipic acid than wild-type (Fig. 6c). Finally, higher ^14^C-DC_12_ oxidation was observed in Sirt5KO MEFs (Fig. 6d). An attempt was made to measure ^14^C-DC_12_ oxidation in liver, but the substrate was found to be unsuitable for assaying broken cells or tissue homogenates. Together these data suggest increased utilization of the ω-oxidation pathway in the absence of Sirt5.

### Loss of mitochondrial C_12_ FAO causes periportal macrovesicular steatosis on coconut oil-diet

**Figure 7:**
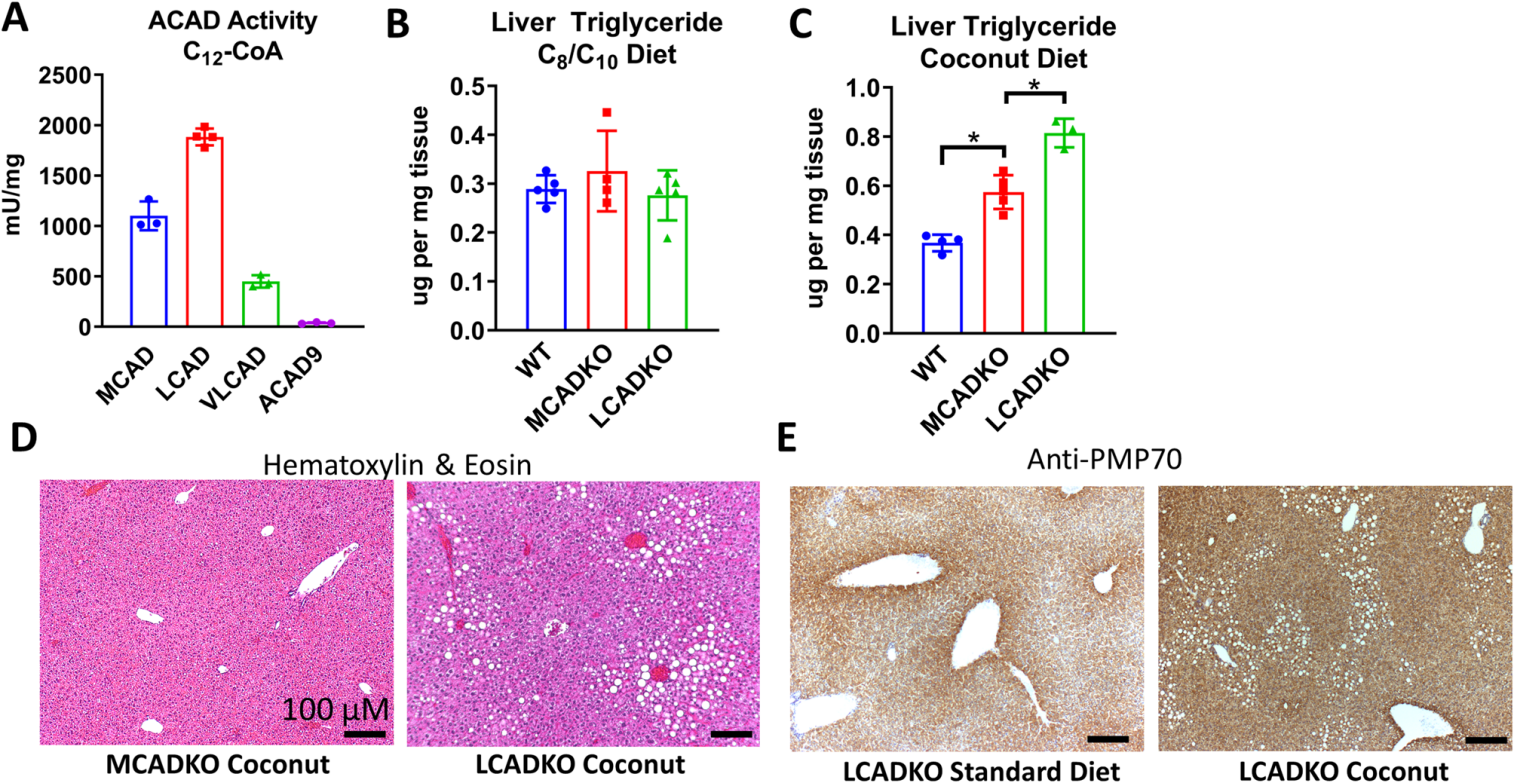
*Loss of mitochondrial FAO causes periportal macrovesicular steatosis on coconut oil diet. (A)* Specific activities of four recombinant mitochondrial acyl-CoA dehydrogenases with C_12_-CoA as substrate indicates LCAD and MCAD are the predominant C_12_-utilizing enzymes. **(B,C)** Liver triglyceride content following either five weeks of C_8_/C_10_ diet (panel **B**) or coconut oil diet (panel **C**). **(D)** Representative H&E staining of MCADKO and LCADKO liver following five weeks of coconut oil feeding reveals periportal macrovesicular steatosis in LCADKO mice. **(E)** Representative immunostaining for the peroxisomal marker PMP70 in LCADKO mice on either standard diet or coconut-oil diet for five weeks. Scale bar, 100 µM.

The observations of normal peroxisomal C_12_ oxidation and elevated ω- oxidation in Sirt5KO mice suggested that the defect in C_12_ metabolism must be mitochondrial. The first step in mitochondrial FAO is catalyzed by the acyl-CoA dehydrogenase (ACAD) enzyme family. There are four ACAD enzymes with partially overlapping substrate specificities^28^. To determine which ACADs contribute to C_12_-CoA metabolism, we compared the specific activities of all four recombinant enzymes with C_12_-CoA. Long-chain acyl-CoA dehydrogenase (LCAD) exhibited the highest specific activity with C_12_-CoA, followed by MCAD (Fig. 7a). Very long-chain acyl-CoA dehydrogenase (VLCAD) had low activity with C_12_-CoA and ACAD9 activity was negligible.

To test whether loss of mitochondrial C_12_ FAO capacity is sufficient to recapitulate the macrovesicular steatosis phenotype, we maintained MCADKO and LCADKO mice on the various diets for five weeks. On standard diet, neither knockout strain exhibited visible macrovesicular steatosis upon H& E staining of liver tissue (data not shown). Surprisingly, while MCAD is the predominant ACAD enzyme metabolizing C_8_-CoA and C_10_-CoA, maintaining MCADKO mice on the high-fat C_8_/C_10_ diet did not result in increased triglyceride storage (Fig. 7b) or any signs of macrovesicular steatosis following histological staining (data not shown). With the coconut oil diet, both MCADKO and LCADKO mice showed significantly increased liver triglycerides (Fig. 7c), but only LCADKO livers exhibited the periportal macrovesicular steatosis phenotype (Fig. 7d). Immunostaining for PMP70 in liver from LCADKO fed coconut oil also revealed a pan-zonal distribution of peroxisomes similar to that observed in Sirt5KO liver (Fig. 7e).

### Sirt5KO liver shows reduced activation of C_12_ to C_12_-CoA

**Figure 8:**
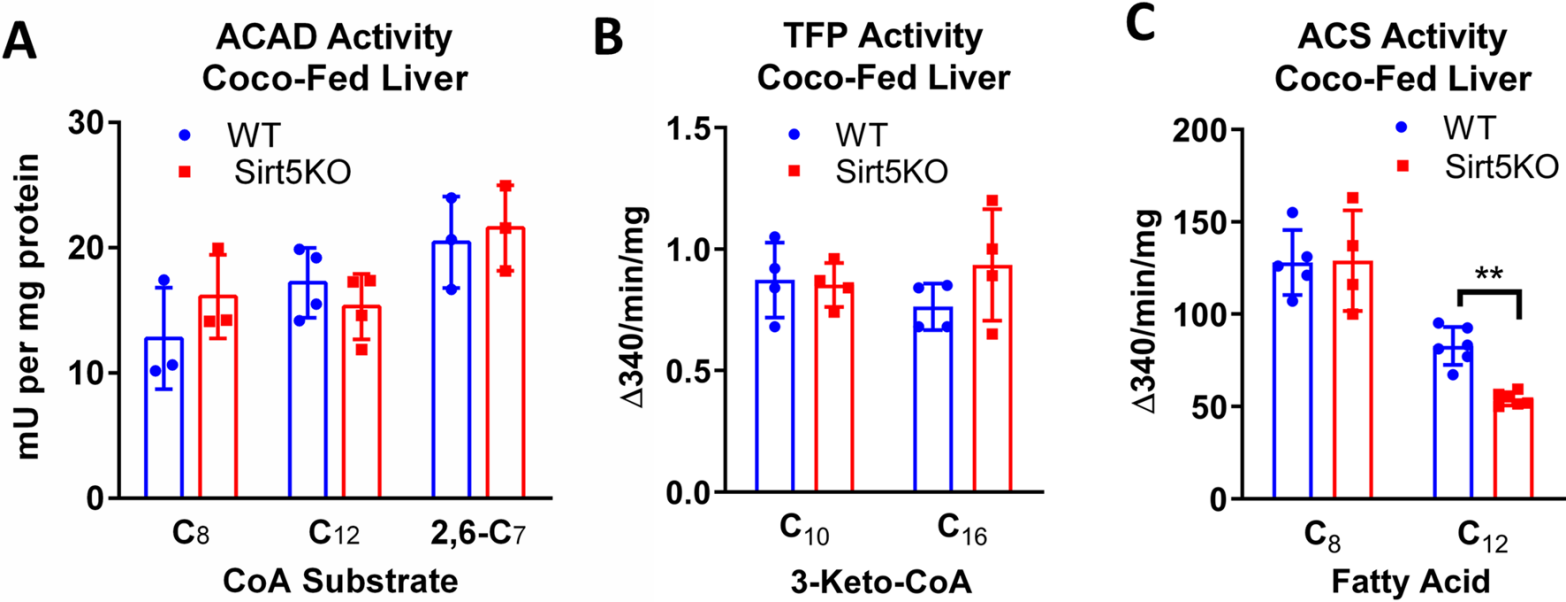
Sirt5KO liver shows reduced activation of C_12_ to C_12_-CoA. N = 3–5 liver homogenates from coconut-fed wildtype (WT) and Sirt5KO mice were tested for acyl-CoA dehydrogenase (ACAD) enzymatic activity with MCAD and LCAD substrates (panel **A**), mitochondrial trifunctional protein (TFP) activity with medium and long-chain substrates (panel **B**), and mitochondrial acyl-CoA synthetase (ACS) activity with C_8_ and C_12_ fatty acids (panel **C**). **P < 0.01.

The experiments described above point to a mitochondrial FAO defect in Sirt5KO livers that causes reduced C_12_ oxidation but not C_8_ oxidation. C_12_ is the transition point between medium-chain and long-chain fatty acids; it is utilized primarily by the long-chain FAO machinery while C_8_ is the optimal chain length for the medium-chain FAO machinery. We therefore hypothesized that reduced function of either LCAD or trifunctional protein (TFP), which together catalyze the four reactions in the long-chain FAO cycle, may explain the C_12_-specific phenotype. Both LCAD and TFP are heavily acylated enzymes with multiple Sirt5-targeted lysine residues^3^, but their activity has not heretofore been measured in Sirt5KO liver. First, the ETF fluorescence reduction assay was used to measure acyl-CoA dehydrogenase activity in liver lysates from coconut-oil fed wild-type and Sirt5KO mice. Three substrates were tested—C_8_-CoA, which is specific for MCAD; C_12_-CoA, which is primarily metabolized by LCAD with overlapping activity from MCAD and very long-chain acyl-CoA dehydrogenase (VLCAD); and 2,6-C_7_-CoA, a branched-chain substrate specific for LCAD^29^. There was no statistically significant change in acyl-CoA dehydrogenase activity in Sirt5KO livers with any of the three substrates (Fig. 8a). Next, we measured activity of TFP with both a medium-chain substrate (3-keto-C_10_-CoA) and a long-chain substrate (3-keto-C_16_-CoA). As with LCAD, no significant change in TFP activity was observed (Fig. 8b).

Another difference in substrate handling between C_12_ and C_8_ is at the acyl-CoA synthetase step, which activates fatty acids to acyl-CoAs prior to FAO. C_8_ more readily diffuses through membranes than C_12_ and is activated to C_8_-CoA inside the mitochondrial matrix by the ACSMs. C_12_ is a poor substrate for the ACSMs^13^. This suggests that C_12_ is activated by members of the ACSL family. There are no ACSLs in the matrix; rather, they are localized to the outer mitochondrial membrane facing the cytosol as well as on the ER membrane^14^. To determine whether loss of Sirt5 alters activity of the acyl-CoA synthetases, we isolated liver mitochondria and measured conversion rates of ^14^C–C_8_ and ^14^C–C_12_ to their respective acyl-CoAs. The rate of mitochondrial ^14^C–C_8_ activation was not different between coconut-fed wild-type and Sirt5KO livers while the rate of mitochondrial ^14^C–C_12_ activation was significantly lower (Fig. 8c).

## Discussion

In the nutritional supplement industry, the term MCT is used a catch-all for coconut oil, pure C_8,_ and mixed C_8_/C_10_ products. Our data indicate that these products may be metabolized very differently. C_8_/C_10_ feeding resulted in periportal microvesicular steatosis. In mice consuming the C_12_-rich coconut oil diet this effect was greatly exacerbated, with steatosis spread throughout the liver. Loss of either Sirt5 or the mitochondrial FAO enzyme LCAD pushed the phenotype into macrovesicular steatosis in the periportal zone. Loss of MCAD, a mitochondrial FAO enzyme with high specificity for C_8_-CoA/C_10_-CoA, did not increase triglyceride storage over wildtype levels or cause macrovesicular steatosis on the high C_8_/C_10_ diet. Thus, stressing mice partially deficient in mitochondrial C_12_ FAO (Sirt5KO, LCADKO) with a diet rich in C_12_ is a combination sufficient to produce periportal macrovesicular steatosis, while stressing mice deficient in C_8_/C_10_ FAO (MCADKO) with a diet rich in C_8_/C_10_ does not produce this phenotype. We postulate that this is because C_12_ is metabolized differently than fatty acids that are just 2–4 carbons shorter.

Medium-chain fatty acids are often stated to enter mitochondria by diffusion and rapidly undergo β-oxidation^7^. C_8_, the prototypical medium-chain fatty acid, is well known to enter mitochondria independently of the carnitine transport system^30,31^. Etomoxir, an irreversible inhibitor of the key transport enzyme carnitine palmitoyltransferase-1 (Cpt1), is completely without effect on liver C_8_ oxidation^32^. C_12_ has been poorly studied in comparison to C_8_. In our experiments, etomoxir blocked C_12_ oxidation by 33% but C_16_ oxidation by 86% (Fig. 5). This indicates that C_12_ partly behaves like a long-chain fatty acid in regard to mitochondrial membrane transport. Perhaps of greater significance is the difference between C_8_ and C_12_ in terms of the mechanism of activation to CoA. We observed normal C_8_-CoA synthetase activity in Sirt5KO liver but significantly reduced C_12_-CoA synthetase activity (Fig. 8). This data suggests that the two chain lengths are not activated to CoA by the same enzyme. Liver abundantly expresses two isoforms of medium-chain acyl-CoA synthetase known as ACSM1 and ACSM2. ACSM1 and ACSM2 are localized to the mitochondrial matrix where they activate free medium-chain fatty acids into medium-chain acyl-CoAs for β-oxidation. Vessey et al^13^ purified ACSM1 and ACSM2 from human liver mitochondria and characterized reactivity against a range of fatty acid substrates. Interestingly, ACSM1 is 40-fold less active with C_12_ than with C_8_, while ACSM2 is 90-fold less active with C_12_. Thus, if these enzymes are tasked with activating C_12_ that has diffused into the mitochondria, the reaction rate would be predicted to be slow. In contrast, the long-chain acyl-CoA synthetase (ACSL) enzyme family, of which there are four isoforms expressed in liver, exhibit high reactivity with C_12_ and low reactivity with C_8_^33,34^. ACSLs are not present in the matrix, but rather are inserted into the outer mitochondrial membrane, ER membrane, and plasma membrane, facing into the cytosol^14^. We previously found multiple Sirt5-targeted lysine residues on ACSL1 and ACSL5^35^, but no Sirt5 target sites have been identified on ACSL3 or ACSL4, which are also expressed in liver. Each ACSL activates fatty acids to acyl-CoAs for a different metabolic purpose. For example, ACSL1 is thought to preferentially channel acyl-CoAs into the mitochondria, while ACSL3 has been implicated in lipogenesis^14^. We postulate that decreased activity of ACSL1, but normal activity of ACSL3, could cause partitioning of fatty acids like C_12_ into lipid droplets rather than into mitochondria in Sirt5KO liver.

Another key difference between exogenous C_8_ and C_12_ fatty acids is the ability of the latter to be metabolized partially by peroxisomal β-oxidation. Peroxisomes were previously shown to be more abundant in the pericentral zone, which our PMP70 immunostaining confirms^36^. Interestingly, coconut oil feeding appeared to recruit peroxisomes into the periportal zone, such that the anti-PMP70 staining became azonal. PMP70 staining appeared stronger in coconut-fed Sirt5KO liver compared to wild-type (Fig. 5e) yet immunoblotting for the peroxisomal enzyme ACOX1 revealed a trend for lower ACOX1 in Sirt5KO liver (Fig. 5c,d). This apparent discrepancy between peroxisomal abundance (PMP70 staining) and peroxisomal enzyme content (ACOX1) has been reported previously in animals treated with peroxisome-proliferator activated receptor-α (PPARα) agonist drugs and may reflect newly formed peroxisomes that have membrane marker proteins like PMP70 but are largely devoid of peroxisomal matrix proteins^37^. Future work will address whether such a phenomenon may be occurring in Sirt5KO liver with coconut oil feeding. It must also be tested whether C12 can alter the distribution of peroxisomes in human hepatocytes. Peroxisomal biogenesis and the peroxisomal FAO pathway are well known to be more inducible in rodents than in humans, possibly due to the much higher abundance of PPARα in rodent liver^38,39^.

While peroxisomes have the capacity to metabolize ^14^C–C_12_ in liver homogenates when mitochondria are inhibited with KCN, it is not currently possible to determine the relative disposition of C_12_ through peroxisomes versus mitochondria in vivo*.* What is clear is that increased peroxisome abundance does not prevent lipid accumulation or macrovesicular steatosis in either Sirt5KO or LCADKO livers. Peroxisomes are known to interact directly with lipid droplets^40^. Elimination of some peroxisomal membrane “Pex” proteins results in reduced lipid droplet size, thought to be due to shared biogenesis mechanisms between peroxisomes and lipid droplets^41^. Another interesting possibility requiring future investigation is that periportal peroxisomes in Sirt5KO and LCADKO livers are partially chain-shortening excess C_12_ to acetyl-CoA, which is being released to the cytosol and used for fatty acid synthesis. Intriguingly, the peroxisomal membrane protein PMP70 physically interacts with fatty acid synthase^42^. Further, experiments with ^13^C-fatty acid tracers showed significant labeling of cytosolic malonyl-CoA, consistent with peroxisomally-produced acetyl-CoA being converted to malonyl-CoA in the cytosol^43,44^. This would not only provide building blocks for fatty acid synthesis, but also further inhibit mitochondrial FAO at the level of Cpt1, leading to a vicious cycle that promotes steatosis.

In summary, this work shows that MCT, and particularly C_12_-rich coconut oil, is associated with a strongly-zoned pattern of lipid accumulation exacerbated by impaired mitochondrial FAO. People consuming MCT as a dietary supplement are not likely to consume the quantity of MCT that the animals did in our experiments. However, preterm infants, patients with epilepsy and FAO disorders, and other patient groups for whom long-chain fatty acids are contraindicated may consume 40% or more of their calories from MCT. It remains to be seen whether long-term consumption of MCT in these patients leads to NAFLD or other hepatic complications. Finally, it is of note that the periportal macrovesicular steatosis seen in Sirt5KO and LCADKO mice consuming coconut oil resembles pediatric NAFLD^23^. For reasons that are not understood, adult NAFLD begins in the pericentral zones and slowly spreads towards the periportal zones as the disease progresses, while pediatric NAFLD shows the opposite pattern. It is tempting to speculate that impaired FAO may contribute to the development of pediatric NAFLD. The biological roles of both Sirt5 and LCAD in humans are poorly understood. LCAD exhibits a much more restricted expression pattern in human than in rodents^45,46^. LCAD is, however, expressed and active in human liver^47^. Our results here suggest it could play a role in the hepatic response to dietary medium-chain fatty acids. Further, common polymorphisms exist in both the Sirt5 and LCAD genes that may affect expression/activity of these enzymes^48,49^. In any case, the consumption of coconut oil, which is increasingly being incorporated into processed foods, may be contraindicated for children with other known NAFLD risk factors.

## Experimental procedures

### Animals experimentation

All animal protocols were approved by the University of Pittsburgh Institutional Animal Care and Use Committee (IACUC), and all experiments were conducted in accordance with the guidelines and regulations set forth in the Animal Welfare Act (AWA) and PHS Policy on Humane Care and Use of Laboratory Animals. SIRT5−/− and wildtype control mice were purchased from Jackson Laboratories (Bar Harbor, ME) and bred in-house. LCAD−/− mice and medium-chain acyl-CoA dehydrogenase (MCAD) −/− mice were obtained from the Mutant Mouse Regional Resource Center. All strains are on a mixed background of C57Bl/6 and 129. High-fat diets based on coconut oil (D12331), C_8_/C_10_ (D17011004; 60% C_8_ and 40% C_10_), or lard (D12451) were purchased from Research Diets, Inc. Mice were placed onto the high-fat diets for five weeks beginning at age 6–8 weeks. Both genders were used; the gender for a given experiment is specified in “Results”. All tissues were collected in non-fasted animals between 9:00 and 11:00 a.m. Euthanasia was conducted using inhaled CO_2_ gas according to IACUC recommendations.

### Fatty acid oxidation assays

^14^C-labeled lauric acid (C_12_) and palmitic acid (C_16_) were from PerkinElmer, while ^14^C-octanoic (C_8_) and dodecanedioc (dicarboxylic C_12_) acids were from Moravek, Inc. All but C_8_ were bound to fatty acid-free albumin; C_8_ was dissolved in 10 mg/ml α-cyclodextrin. For experiments with Sirt5 knockout mouse embryonic fibroblasts (MEFs), which were a kind gift of Dr. Eric Verdin (Buck Institute), the cells were grown to near confluence in T75 flasks. The cells were harvested and resuspended in DMEM containing 5 mM glucose and 125 µM of labeled fatty acid. Cells were rotated in a 37 °C water bath for 1 h. Then, perchloric acid was added to a final concentration of 0.5 M and ^14^CO_2_ was evolved, captured, and counted as described^50^. FAO in liver lysates were conducted in similar fashion. Freshly isolated liver was weighed and homogenized in 10 volumes of Mir05 media^51^. The lysate was centrifuged at 500 × *g* for 5 min to remove nuclei and unbroken cells. FAO reactions consisted of 5 µl of lysate in a total reaction volume of 200 µl containing 100 mM sucrose, 10 mM Tris–HCl, 5 mM KH_2_PO_4_, 0.2 mM EDTA, 0.3% fatty acid-free BSA, 80 mM KCl, 1 mM MgCl_2_, 0.2 mM l-carnitine, 0.1 mM malate, 0.05 mM coenzyme A, 2 mM ATP, 1 mM DTT, and 125 µM of labeled fatty acid. In some experiments, peroxisomal FAO was measured by inhibiting mitochondria with either 2 mM freshly prepared KCN or by preincubation for 15 min with 100 µM etomoxir. ^14^C-labeled FAO products were separated from the reactions by extraction with chloroform/methanol and counted.

### Immunoblotting

Western blotting was performed after electrophoresis on Criterion SDS polyacrylamide gels (BioRad, Hercules, CA) and transfer to nitrocellulose membranes. Antibodies used were: rabbit anti-succinyllysine (PTM Biolabs), anti-malonyllysine (PTM Biolabs), anti-acyl-CoA oxidase-1 (ACOX1; Abcam), anti-enoyl-CoA hydratase/3-hydroxyacyl-CoA dehydrogenase (EHHADH; Abcam), and anti-β-actin (Proteintech). After incubation with HRP-conjugated secondary antibodies (1:5000) blots were visualized with chemiluminescence. In some experiments the blots were scanned and subjected to densitometric analysis using ImageJ software.

### Histology and immunohistochemistry

Fresh portions of liver were fixed in 4% paraformaldehyde and embedded in paraffin, sectioned at 4 μm, and stained with hematoxylin and eosin (H&E) using standard methodology. Another portion of liver was embedded in OCT. frozen, and sectioned at 5–10 μm thick for Oil-Red-O staining. Immunostaining was performed as previously described^50,52^ using anti-peroxisomal membrane protein-70 (PMP70; Abcam ab85550 at 1:200) and anti-glutamine synthase (GS; Sigma G2781 at 1:1500). Assessment of histology was performed by the Biospecimen Repository and Processing Core of the Pittsburgh Liver Research Center.

### Urine adipic acid

Urine was collected from experimental and control animals over a 24 h period using metabolic caging. Creatinine concentration was determined using a kit (Cayman Chemicals, Inc). Adipate was determined in the context of standard clinical urine organic acid assessment at the Children's Hospital of Pittsburgh Clinical Biochemical Genetics Laboratory^53,54^. Briefly, a volume of urine was utilized equal to 1.0 mM creatinine. A 2-phenylbutyrate internal standard is included. Organic acids were extracted by sequential ethyl ether and acetoacetate extractions. Trimethylsilane derivatization was employed. Analysis utilized Agilent 7890A gas chromatography and 5975C mass spectrometry. All peak areas were normalized to that of 2-phenylbutyrate. Fragmentation patterns of urine analytes were compared to an internally compiled fragmentation library and the fragmentation library of the National Institute for Standards and Technology.

### Liver triglyceride content

Approximately 100 mg of liver was weighed, chopped finely and digested in 350 µl of ethanolic KOH at 55 °C overnight. After centrifugation to pellet any undigestible material, the sample volumes were brought to 1.2 ml with 50% EtOH. 200 µl was removed to a new tube and mixed with 215 µl of 1 M MgCl_2_. Samples were clarified once more by centrifugation at 8000 × g and the supernatant used for assaying glycerol content. Glycerol was assayed spectrophotometrically at 540 nm using a kit (Sigma). Triglyceride content was normalized to tissue weight.

### Enzyme activity assays

The anaerobic electron transfer flavoprotein (ETF) fluorescence reduction assay was used to measure acyl-CoA dehydrogenase activities as described^55^. His-tagged recombinant enzymes were purified as described ^47^. Assays contained either 150 ng of purified recombinant protein or 200 µg of total liver protein, 2 µM recombinant porcine ETF, and 25 µM acyl-CoA substrate. C_8_ and C_12_ acyl-CoAs were from Sigma (St. Louis, MO) and 2,6-dimethylheptanoyl-CoA was from Toronto Research Chemicals, (Toronto, ON). Reduction of ETF fluorescence was followed for 1 min and used to calculate specific activity normalized to protein concentration. Mitochondrial trifunctional protein (TFP) activity was measured in the reverse direction using 3-ketodecanoyl-CoA (3-keto C_10_-CoA) and 3-ketopalmitoyl-CoA (3-keto-C_16_-CoA). Reactions contained 30 µg of liver homogenate and 50 µM substrate in a reaction buffer consisting of 100 mM KPO_4_, 50 mM MOPS, 0.1 mM DTT, 0.1% Triton-X100, and 0.15 mM NADH. The conversion of NADH to NAD^+^ was followed in a plate reader at 340 nm. C_8_ and C_12_-CoA synthetase activities were measured using ^14^C-C_8_ and ^14^C-C_12_. Liver mitochondria were isolated by differential centrifugation and resuspended in cold SET buffer (10 mM Tris–HCl, 250 mM sucrose, 1 mM EDTA). Synthetase reactions (200 µl) contained 5 µl of homogenate with 10 µM ^14^C-fatty acid in a buffer of 40 mM Tris–HCl, 5 mM ATP, 5 mM MgCl_2_, 4 mM CoA, 0.8 mg/ml Triton WR1339, and 1 unit/ml inorganic pyrophosphatase. After 2 min incubation at 37 °C, reactions were stopped with sulfuric acid and extracted either four times with ether (C_8_-CoA synthetase) or chloroform/methanol (C_12_-CoA synthetase) to separate formed acyl-CoAs from excess ^14^C-fatty acid. Activities were normalized to protein concentration.

## Supplementary information

Supplementary Information.
