# Supplementary material for: Impaired mitochondrial medium-chain fatty acid oxidation drives periportal macrovesicular steatosis in sirtuin-5 knockout mice
Source: Sci Rep. 2020 Oct 27;10:18367. doi: 10.1038/s41598-020-75615-3 (PMC7591893; doi:10.1038/s41598-020-75615-3)
Supplement: Supplementary file 1 — Supplementary Information. [file 41598_2020_75615_MOESM1_ESM.pdf]

Impaired mitochondrial medium-chain fatty acid oxidation drives periportal macrovesicular steatosis in sirtuin-5 knockout mice

Eric S. Goetzman[1,4*], Sivakama S. Bharathi[1], Yuxun Zhang[1], Xue-Jun Zhao[1], Steven F. Dobrowolski[2], Kevin Peasley[1], Sunder Sims-Lucas[1], and Satdarshan P. Monga[3,4]

[1]Department of Pediatrics, University of Pittsburgh School of Medicine, University of Pittsburgh, Children's Hospital of Pittsburgh of UPMC, Pittsburgh, PA, USA

[2]Department of Pathology, University of Pittsburgh School of Medicine, Pittsburgh, PA, USA

[3]Division of Experimental Pathology, Department of Pathology and Division of Gastroenterology, Hepatology and Nutrition, Department of Medicine, University of Pittsburgh School of Medicine, Pittsburgh, PA, USA

[4]Pittsburgh Liver Research Center, University of Pittsburgh Medical Center and University of Pittsburgh School of Medicine, Pittsburgh, PA, USA

*Corresponding author: Eric Goetzman

Email: eric.goetzman@chp.edu

Supplemental Figure 1

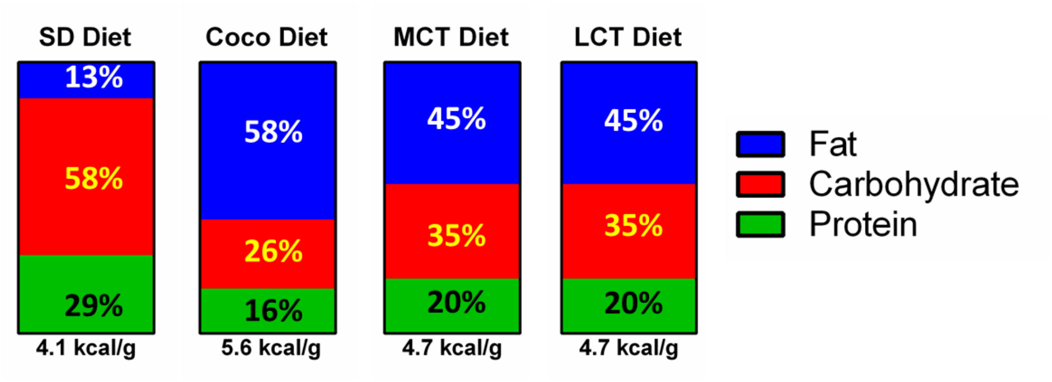

Relative composition of four diets used in the study. The high-fat coconut-oil diet (D12331), C_8/C_{10} medium-chain triglyceride (MCT) diet (D17011004) which contains lipids from fractionated coconut oil, and the traditional long-chain triglyceride (LCT) diet based on lard (D12451) were all purchased from Research Diets, Inc. and fed for a period of 5 weeks.

Supplemental Figure 2

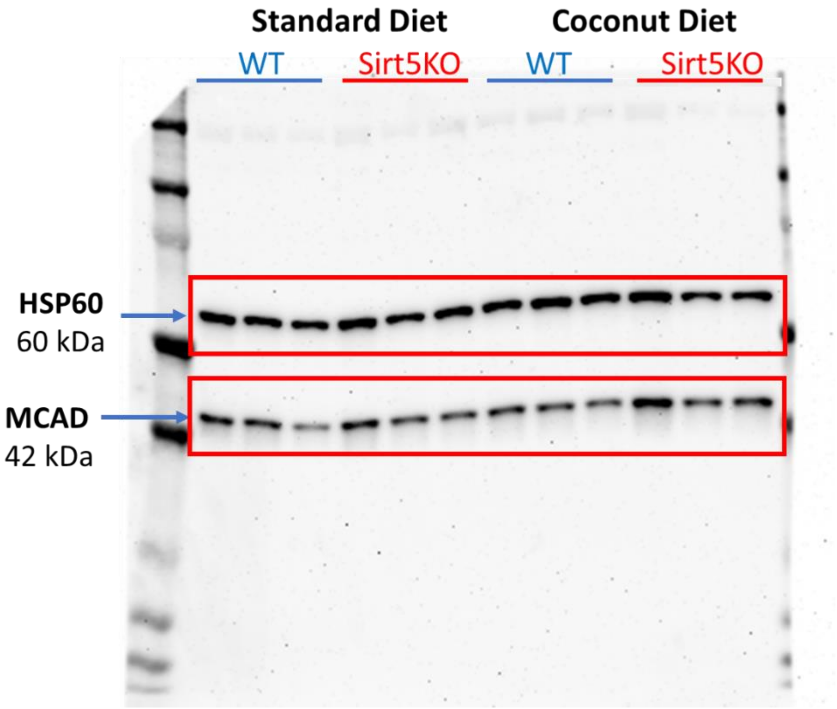

Full-length western blot for MCAD and Hsp60 related to Figure 4. Membrane was incubated with antibodies against MCAD and Hsp60 (mitochondrial marker as loading control) simultaneously.

Supplemental Figure 3

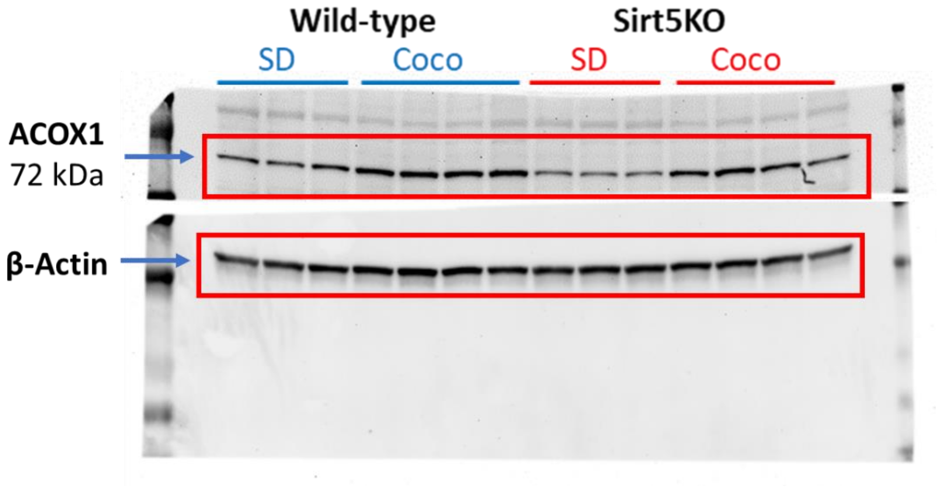

Uncropped blots related to Figure 5. The membrane was cut horizontally just above the 50 kDa marker and the top piece immunoblotted for ACOX1 while the bottom piece was immunoblotted for β-actin as a loading control.

Supplemental Figure 4.

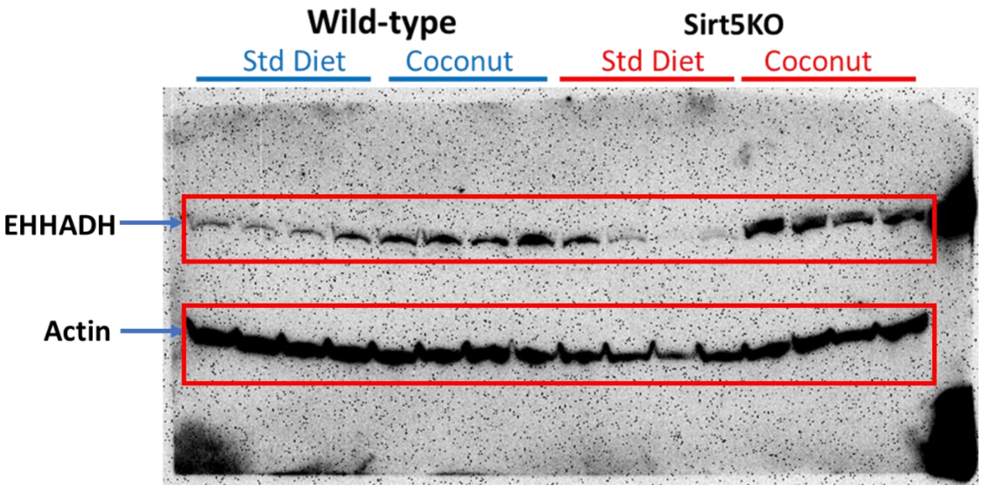

Uncropped blot related to Figure 6. The membrane was incubated with anti-EHHADH and anti-β-actin antibodies simultaneously.
